# Manual and Application-Based Carbohydrate Counting and Glycemic Control in Type 1 Diabetes Subjects: A Narrative Review

**DOI:** 10.3390/healthcare11070934

**Published:** 2023-03-24

**Authors:** Sara A. AlBabtain, Nora O. AlAfif, Dara AlDisi, Saad H. AlZahrani

**Affiliations:** 1 Clinical Nutrition Administration, King Fahad Medical City, Riyadh Second Health Cluster, Riyadh 11525, Saudi Arabia; 2 Department of Community Health Sciences, College of Applied Medical Sciences, King Saud University, Riyadh 11433, Saudi Arabia; 3 Obesity, Endocrine and Metabolism Center, King Fahad Medical City, Riyadh 11525, Saudi Arabia

**Keywords:** type 1 diabetes mellitus, hemoglobin A1c, carbohydrate counting, carbohydrate mobile app, diabetes nutrition

## Abstract

Type 1 diabetes (T1DM) is the most common chronic disease in young adults and children, which is treated with insulin, usually given as basal and boluses. Carbohydrate counting (CHOC) helps patients to determine the correct meal doses. The aim of this review is to study the effect of CHOC on glucose control, body weight, insulin dose and quality of life (QoL). The literature search was conducted using PubMed from January 2010 to October 2022. Studies included in this review are limited to randomized controlled studies involving an intervention group undergoing CHOC and a control group following the usual practice, measuring glycosylated hemoglobin (HbA1c) as a parameter of glucose control and involving only T1DM subjects. A total of ten articles were found to fulfill the criteria involving 1034 patients. Most of the studies showed a positive impact of CHOC on glucose control, especially in adults, where five out of six studies were statistically positive. However, in pediatrics, only two out of four showed a positive outcome. In all four studies using mobile applications, CHOC was better at controlling glucose. No difference was seen between the CHOC group and the control regarding the risk of severe hypoglycemia. In fact, two studies have shown lower hypoglycemia rates. No change in weight was observed in most of the studies (six out of eight). In subjects with T1DM, CHOC might provide better glucose control than traditional care without a significant increment in severe hypoglycemia or weight gain. Mobile application-based models showed promising results in glucose control.

## 1. Introduction

Type 1 diabetes mellitus (T1DM) is the most common chronic disorder globally. T1DM accounts for about 2% of diabetes cases globally, nearly affecting nine million subjects [1]. Where in the kingdom of Saudi Arabia (KSA), it is about 31.5/100 k, putting our country in the fifth rank internationally [2]. T1DM is an autoimmune disease characterized by the destruction of insulin-producing cells in the pancreas, resulting in insufficient production of insulin and, subsequently, higher glucose levels [3]. It is assumed that an interplay between genetic and environmental factors might trigger the immune system to attack beta cells [4]. The strongest genetic predisposition found is the presence of a polymorphism at class II human leukocyte antigen genes encoding DQ and DR [5].

Uncontrolled T1DM is associated with multiple macro- and micro-vascular complications in both adults and children, which is determined by the degree and duration of diabetes [6]. If large vessels are affected, ischemic events might occur, causing ischemic heart disease or cerebrovascular disease, and if small vessels are affected, nephropathy, retinopathy, or retinopathy, among many other complications, might occur [7]. Insulin is used for glycemic control as well as a balanced diet and regular physical activity in the therapy of T1DM. Patients’ daily insulin requirements vary according to their age, diet, self-monitoring of blood glucose, and daily routines [8].

Carbohydrate counting (CHOC), one of the varied ways of estimating insulin doses, is considered the most efficient method of calculating insulin doses associated with meals [9]. This method allows greater flexibility in diet and, in some cases, can reduce the burden of diabetes [10]. It is the primary macronutrient that influences postprandial glucose levels. The insulin dose could be adjusted in relation to carbohydrate intake in order to improve glycemic control and quality of life (QoL) [11].

CHOC helps subjects with T1DM to adjust insulin doses to the carbohydrate content in a meal. Patients are usually taught the carbohydrate component of each type of food and the insulin-to-carbohydrate ratio, which is calculated and adjusted by a clinical dietician [12].

In the first half of the 20th century, CHOC was developed as a meal-planning technique for calculating insulin doses based on the total amount of carbohydrates consumed during each meal [13]. CHOC can be divided into three types based on the level of complexity. At Level 1, or basic, patients are taught about CHOC and carbohydrate consistency. At Level 2, or intermittent, participants learn about the relationship between food, diabetes medication, physical activity, and blood glucose levels. They also learn about the steps needed to manage these variables based on blood glucose level patterns. At Level 3, or advanced, which is discussed in this review, patients with T1DM using multiple daily injections or insulin infusion pumps understand how to match short-acting insulin to carbohydrates using carbohydrate-to-insulin ratios. The three levels emphasize the importance of portion control [14,15].

There are two methods for calculating CHOC: listing carbohydrate equivalents (A) and measuring carbohydrates in grams (B). Method A categorizes foods into portions of 15 g of carbohydrate each to determine whether they are equivalent. The second method involves summarizing the number of carbohydrate grams that are present in each food per meal based on information provided on food labels and tables [8].

Carbohydrate counting was popularized by the Diabetes Control and Complications Trial (DCCT) [10]. In this study, patients were randomized to either receive intensive insulin or conventional treatment. Intensive treatment was associated with lower microvascular complication rates. Moreover, CHOC use was linked with better glucose control. In a clinical study of 51 adults with T1DM, glycosylated hemoglobin (HbA1c) decreased by 0.5% in people who adjusted their pre-meal insulin doses according to CHOC compared with those who used a fixed dose [16]. In another study of 55 adult patients with T1DM, Dias and colleagues demonstrated that HbA1c levels decreased despite an increase in the total daily insulin dose without any weight gain [17]. Furthermore, the Dose Adjustment for Normal Eating (DAFNE) Study Group studied the effect of a structured dietary training program on T1DM subjects over a period of six months. In this study, subjects were divided into an immediate training group receiving the course instantly (immediate DAFNE) to a delayed group, who initially served as controls (delayed DAFNE). HbA1c in the immediate DAFNE patients was significantly lower than that of delayed DAFNE patients. Furthermore, improvements in the impact of diabetes on dietary freedom and QoL was observed [18].

Diabetes digital health is rapidly evolving and provides great flexibility and better guidance for diabetic individuals [19,20]. In the nutrition field, CHOC and bolus calculator apps are widely available to help patients for better estimation of dietary carbohydrate contents and, consequently, better estimation of insulin doses [21,22].

This narrative review aimed to evaluate and appraise the existing evidence for the role of manual and mobile applications-based CHOC in reducing glucose levels in subjects with T1DM as compared to standard care or other forms of dietary advice. Furthermore, in this work, the safety and impact of CHCO on weight and quality of life were assessed as well. 

## 2. Materials and Methods

**Figure 1:**
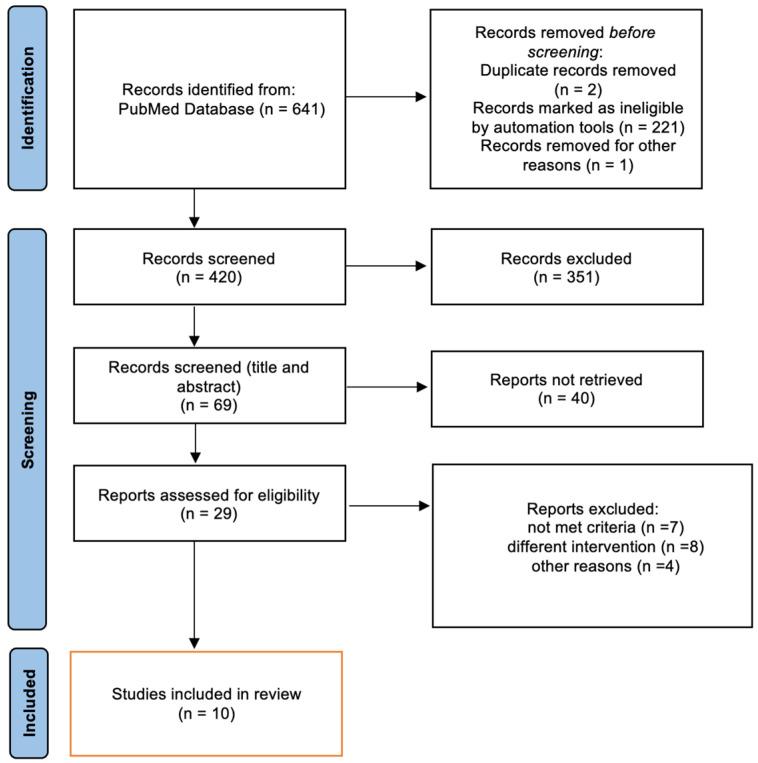
The flow diagram of the literature review process.

Search strategy: A database search was conducted between January 2010 and October 2022 using PubMed. The database was searched with three keywords, the first representing “diabetes type 1” (including IDDM, T1DM), the second representing “carbohydrate counting” (including carbohydrate, carb count), and the third representing “application” (including software, mobile, app). In order to link the keywords, we used the Boolean operators “OR” (for words that describe a single component) and “AND” (for words that describe both components). The search was limited to articles including human subjects and published in the English language. This review was reported according to the PRISMA statement [23] (Figure 1).

Eligibility criteria: Outcomes were evaluated in patients who were randomly assigned to intervention or comparison groups. As described below, data were analyzed in the context of patient characteristics, interventions, comparisons, and outcomes (PICO). Patients were T1DM adults, children, and adolescents on continuous subcutaneous insulin infusions (CSII) or multiple daily insulin injections for at least three months. Pregnant women and lactating subjects with serious diabetes complications were excluded. Intervention: in the intervention group, participants were manually trained on advanced CHOC.

Participants used an app to calculate how much insulin they would require before each main meal. Comparison: individuals in the comparison group received traditional nutritional advice and used fixed doses of fast or regular insulin before meals. Outcome: the outcomes assessed were a reduction in HbA1c and severe hypoglycemia, an improvement in QoL, a gain in weight or body mass index (BMI), and safety for the CHOC application (app) and insulin dose. QoL was assessed using validated questionnaires: the Audit of Diabetes-Dependent Quality of Life (ADDQoL), the Diabetes Treatment Satisfaction Questionnaire (DTSQ), and the Diabetes Quality of Life Measure (DQoL).

Data extraction: the included studies’ data were extracted and entered into a spreadsheet. The following information was obtained: first author, year of publication, country of origin, number of patients, intervention, control, outcomes data HbA1c (%), insulin dose (U/kg), BMI (kg/m^2^) or body weight, follow-up, and severe hypoglycemia episodes and application name if available. The primary outcome was the change in HbA1c concentration, and secondary outcomes were the change in insulin dose, body weight gain or BMI, severe hypoglycemia episodes, QoL, and CHOC app safety.

## 3. Results and Discussion

In this narrative review, ten papers were found eligible according to the prespecified criteria (Figure 1). A total of 751 adults (>18 years) and 283 children and adolescents who were diagnosed with T1DM with a duration of at least three months were included. All studies included were randomized controlled trials in design with a minimum follow-up of at least three months and up to thirty months. Six studies enrolled were conducted among adults, and the remaining four studies included children and adolescents. The detailed characteristics and designs of these studies are summarized in Table 1.

Only one study compared the number of carbohydrates, energy, fat, and fiber intake in the study groups with minor changes after intervention [24].

**Table 1:** Characteristics of studies included in this narrative review.

Author/Year	Country	No. of Patients	Intervention	Control	Result Intervention/Control			Follow-Up
					HbA1c ^1^ % (M ^2^ ± SD ^3^)	Insulin Dose (U/kg ^4^) (M ± SD)	BMI ^5^ (kg/m^2^) (M ± SD) or BW	
**Adult**								
Hommel E. et al., 2016 [25]	Denmark	168	*n* = 84, advanced CHOC using an automated bolus calculator	*n* = 84, advanced CHOC using mental calculations	(8.9 ± 0.7 to 8.4 ± 0.45) (9.0 ± 0.8 to 8.8 ± 0.25)	__	No change in BW	1 year
Schmidt et al., 2012 [16]	Denmark	51	*n* = 21, CarbCount Group: taught CHOC, ICRs, and ISFs were estimated; or *n* = 22, CarbCountABC: taught the same CarbCount group and instructed in the use of the automated bolus calculator (ABC)	*n* = 8, group diabetes education (food recommendations, self-monitoring techniques, and estimated insulin doses)	CarbCount (9.2 ± 0.6 to 8.4 ± 0.9) CarbCountABC (8.8 ± 0.7 to 8.1 ± 0.4) Control (9.10 ± 0.70 to 8.90 ± 1.10)	CarbCount (0.6 ± 0.2 to −0.03 ± 0.11) CarbCountABC (0.7 ± 0.2 to −0.03 ± 0.15) Control (0.7 ± 0.17 to 0.01 ± 0.07)	No change in BW	16 weeks
Laurenzi et al., 2011 [26]	Italy	61	*n* = 28, CHOC education	*n* = 28, usual care	Similar in the two groups (*p* = 0.252)	No changes	BMI 23.7 (21–25.2) at 24 weeks, −0.32 (−0.65 to 0), and 23.8 (20.8–26.8) at 24 weeks, 0.15 (0–0.40)	24 weeks
Scavone et al., 2010 [27]	Italy	256	*n* = 156, CHOC education (4 weeks), reassessed every 3 months	*n* = 73/100, usual care	7.80 ± 1.30 to 7.40 ± 0.90 7.50 ± 0.80 to 7.50 ± 1.10	At the end, 23.5 ± 10.9 vs. 27.7 ± 17.1	No BW gain	9 months
Trento et al., 2011 [28]	Italy	56	*n* = 27, CHOC program (8 sessions), and usual group care	*n* = 29, usual diabetes education and group care	7.60 ± 1.30 to 7.20 ± 0.90 7.70 ± 1.24 to 7.90 ± 1.40	No changes	BMI 24.4 ± 2.6 to 23.4 ± 5.3 23.5 ± 3.3 to 23.5 ± 2.9	30 months
Isaksson S. et al., 2021 [24]	Sweden	159	*n* = 51, food-based approach, and *n*= 52, CHOC	*n* = 55, routine care	FBS 8.1 ± 0.7 to 7.8 ± 0.7 CHOC 7.9 ± 0.7 to 7.8 ± 0.7 RC 8.0 ± 0.7 to 7.9 ± 0.8	No changes	No change in BW	12 months
**Children and adolescents**								
Alfonsi J. et al., 2020 [29]	Canada	46	*n* = 21, CHOC and iSpy app	*n* = 22, CHOC	8.41 ± 1.84 to 8.06 ± 1.43, 8.35 ± 1.32 to 8.80 ± 1.60	__	__	3 months
Goksen et al., 2014 [30]	Turkey	110	*n* = 52, CHOC group	*n* = 32, usual nutritional and diabetic education	8.10 ± 1.00 to 7.87 ± 1.38, 8.43 ± 1.52 to 8.76 ± 1.77	0.92 ± 0.29 to 1.01 ± 0.28, 0.96 ± 0.36 to 1.02 ± 0.31	19.61 ± 3.22 to 20.81 ± 3.38, 20.89 ± 3.31 to 21.80 ± 3.68, and no change	2 years
Enander et al., 2012 [31]	Sweden	40	Group B: *n* = 12, manual CHOC; Group C: *n* = 14, CHOC with a bolus calculator	Group A: *n* = 14, traditional methodology (the plate exchange method)	Group B, 7.7 ± 1.0 to 7.8 ± 0.9; Group C, 7.2 ± 0.6 to 7.6 ± 1.1; and Group A, 7.70 ± 1.00 to 8.00 ± 1.00	Group B, 0.42 ± 0.12 to 0.44 ± 0.14; Group C, 0.45 ± 0.19 to 0.42 ± 0.13; and Group A, 0.43 ± 0.10 to 0.46 ± 0.10	At 12 months: Group C significantly decreased compared with baseline (+1.2 vs. +1.4 kg/m^2^)	12 months
Donzeau A. et al., 2020 [32]	France	87	ACC group: *n* = 40, advanced CHOC	Control group: *n* = 47, standard nutrition	At 3 months, 7.8 ± 0.5 to 7.53 ± 0.61 and 7.8 ± 0.5 to 7.88 ± 0.56; at 12 months, no difference	__	No difference in BMI	52 weeks

^1^ HbAlc: glycosylated hemoglobin; ^2^ mean; ^3^ standard deviation; ^4^ unit per kilogram; and ^5^ body mass index.

Carbohydrate counting education time varies depending on the method of each study depending on the age of the subjects. While individual education was used in some studies individually [16,25,26,29,32], others have used group teaching [24,27,28,30,31], with different durations, starting from two [16,25,30], four [27,31], to eight [24] weeks, or in sessions [26,28,29]. In each sitting, the participants spent an average of three hours, and there was only one study that lasted for ten hours over three days [32].

### 3.1. Primary Outcome

#### Glycemic Control Assessed by HbA1c

In this review, hemoglobin A1c concentration (measures glycemic control within the past three months) is the primary outcome as it is a diabetes mellites control marker most extensively studied and correlated to the risk of complications [33]. Seven out of ten studies showed an improvement in HbA1c in the CHOC arm. Available data for short-term follow-up (less than six months) showed that CHOC is superior for glucose control in four out of five studies [16,24,26,29,32].

In intermediate follow-up studies (9–12 months), only two studies out of five showed an improvement in glucose control with CHOC [24,25,27,31,32]. Isaksson and Donzeau showed an early improvement with CHOC within three months; however, this effect is lost after one year of follow-up [24,32]. In studies conducted over a longer period of time (24–30 months), both studies showed an improvement in HbA1c in subjects following CHOC [28,30]. Goksen et al. showed a positive outcome in a two-year follow-up but not after the first year [30].

The tendency for better control over the short term might be explained by the fact that patients get more compliant and enthusiastic at the start of the study and might lose interest with time. However, this was not the case in all studies, which might be related to the differences in the population and methodology.

Further dividing the studies per age group indicates that adults might gain more benefits in comparison to children and adolescents. Five out of six studies included in this review reported a significant reduction in HbA1c in adult participants [16,25,26,27,28], while two of four studies conducted on children and adolescents observed a reduction in HbA1c levels [29,30]. This variability between adults and children is expected as its more difficult to control the dietary habits in kids as well as the effect of anti-insulin hormones, especially in adolescents [34].

There are some researchers who have reported that CHOC has no benefit compared with simple dietary advice, which was explained by the fact that these studies were conducted on poorly controlled, conventionally treated type 1 diabetic patients who were not given specific insulin adjustment algorithms [14].

### 3.2. Secondary Outcome

#### 3.2.1. Severe Hypoglycemia

Accurate CHOC may be helpful not only in decreasing HbA1c but also in decreasing the incidence of severe hypoglycemic events (≤3 mmol/L) [35]. Reporting of hypoglycemia in this review was variable. While some studies have used a qualitative system, others used a self-reported one [16,25,27,32]. Hommel et al. used a sophisticated system by reporting the duration of time below range in continuous glucose monitoring [25].

Eight out of ten studies have reported hypoglycemia as an outcome. Two studies, Scavone et al. and Trento et al., found that CHOC had resulted in a reduced rate of hypoglycemia episodes compared with controls [27,28]. The other studies did not reveal a significant difference in the rates of hypoglycemia, indicating that CHOC is at least safe, if not beneficial, in lowering low-glucose events. Hypoglycemia outcomes among different studies are summarized in Table 2.

#### 3.2.2. Body Weight

One of the advantages of CHOC is that it provides increased flexibility and diversity when choosing food. In theory, this might lead to increase food intake and, consequently, weight gain in subjects with T1DM who follow CHOC [36].

Nine out of ten studies have reported weight or BMI as an outcome. Seven of them have not demonstrated a difference in weight between the intervention and control group [16,24,25,27,30,31,32], while two studies have shown weight reduction in the CHOC group [26,28]. This finding further strengthens the value of CHOC in controlling DM without major weight gain. However, others have challenged this finding by showing that CHOC was associated with weight gain [36]. This might be explained by the differences in the populations between studies.

#### 3.2.3. Daily Insulin Dose

Daily insulin doses were reported in seven studies out of ten. Most studies were conducted using rapid-acting insulin. Three out of seven studies have shown lower insulin doses in the CHOC arm compared with controls [16,27,31]. Schmidt and colleagues showed a small reduction in the average total daily dose (TDD) in CarbCount and CarbCountABC vs. control (−0.03 ± 0.11, −0.03 ± 0.15 vs. 0.01 ± 0.07 U/kg/day, respectively) [16]. Similarly, Scavone et al. found a reduction in the dose of rapid-acting insulin in the CHOC group vs. control (23.5 ± 10.9 vs. 27.7 ± 17.1 U/day, respectively) [27], while Enander et al. showed only a small reduction in daily basal insulin dose but not in the TDD [31]. On the other hand, three studies have shown no difference in insulin doses [24,26,28], while only one study by Goksen et al. in pediatrics has demonstrated increased insulin doses with CHOC compared with baseline (0.92 ± 0.29 to 1.01 ± 0.28 units/kg/day, respectively) [30]. This controversy might be related to population differences or the methodology used, which is known in the field of medical research. Altogether, CHOC showed a good effect on glucose levels, which was attained without obviously higher insulin doses. Again, this finding comes to favor CHOC and perhaps enhances its safety.

#### 3.2.4. Quality of Life and Satisfaction Questionnaires

Quality of life was reported in six out of ten studies. In these studies, five different questionnaires were used, and heterogeneity in the outcomes was reported [16,24,26,28,29,32]. Four out of six surveys demonstrated improved quality of life in those using CHOC [16,26,28,32]. On the other hand, Schmidt et al. used two questionnaires, DTSQ and ADDQoL, and found different results. The former questionnaire showed improvement in all study arms, with better outcomes in those using CHOC with a bolus calculator, which was explained by the teachings received by all arms. While no differences could be appreciated when using the DTSQ, and again this might be explained by the difference in the sensitivity of the surveys [16].

Donzeau and their group used the Diabetes-specific Quality of Life (DSQOL) for both patients and parents and found a positive impact in the intervention group [32]. Although there was a higher heterogeneity in conducting and reporting the QoL, an obvious trend towards improved QoL with CHOC was noticed.

To conclude, most studies have shown improved quality of life measurements in both children and adults with T1DM.

### 3.3. Carbohydrate-Counting Application Safety

In T1DM management, it is recommended to match insulin doses with carbohydrate intake, blood glucose levels, and level of activity [37]. Bolus insulin should be matched to carbohydrate intake using an insulin-to-carbohydrate ratio (ICR) to achieve optimal blood glucose control after a meal. The insulin sensitivity factor (ISF) indicates whether additional insulin should be added to decrease the blood glucose level to the target range [15]. However, determining an appropriate insulin bolus size several times throughout the day is often challenging and resource-intensive but also crucial [37,38]. Due to these challenges, technologies such as mobile health apps that address these issues have the potential to ease burdens and enhance blood glucose control [29].

Four studies out of ten were conducted using mobile apps. Three used a bolus calculator, and one used the iSpy app. Bolus calculator apps assist patients in calculating the insulin bolus based on the blood glucose value, target blood glucose, ISF, ICR, and others [25], while the iSpy app assists patients with T1DM in CHOC by allowing them to identify the carb content of foods through images [29]. Hommel et al.’s compared mental calculations of CHOC with a bolus calculator in adults for 12 months. The bolus calculator was better at lowering HbA1c [25].

Another work by Schmidt et al. assessed adult participants with T1DM in three groups: on routine diabetic diets, CHOCs, and CHOCs using a bolus calculator for 16 weeks. While no significant difference in HbA1c levels was shown between CHOCs and CHOCs using bolus calculators, treatment satisfaction as measured by DTSQs was significantly greater in CHOCs using a bolus calculator [16].

Additionally, Enander et al. randomized children and adolescents with T1DM into groups: control, manual CHOC, and CHOC with a bolus calculator for one year. In the bolus calculator group, improved postprandial glucose levels were seen by reducing overall and meal-related fluctuations but did not change HbA1c [31].

Alfonsi et al. involved three cycles of iterative usability testing conducted over a period of three months with children and adolescents with T1DM. Participants were randomized into groups of iSpy app users and usual care controls. The iSpy app was linked with improved CHOC accuracy and reduced the frequency of errors, which ultimately resulted in glycemic control [29]. To sum up, mobile applications generally improve the accuracy of CHOC and are associated with better glucose readings, and are going to be used more in the future.

## 4. Limitations

Although this narrative review was conducted using randomized controlled studies, some of them using mobile apps, which of course, will not go without drawbacks. First, the standardization of carbohydrate content in different foods, a variety of populations, and different cultures is a difficult task, especially in foods that do not contain food labels. In fact, this might lead to miscalculation of the actual carbohydrate content, which in turn, might affect the insulin dose and, consequently, the glucose levels. Ultimately, this will lead to considerable heterogenicity in the field of CHOC research, which we have noticed in this work. Although this concern is always there with CHOC; therefore, patients are usually taught to use estimations to overcome this problem. The same concept applied to the use of different CHOC educational methods in different studies, which again might have affected the final outcomes. Having said that, CHOC remains the best tool to adjust insulin doses according to the carbohydrate content, and perhaps technologies such as mobile apps will help to standardize it more. Second, the number of patients recruited in our review is relatively small, especially in children. Third, opposite to expected, in most studies, the QoL in those using CHOC was not superior to those using standard care. This can be partially explained by the differences in populations and the different questionnaires used. Fourth, in all studies, glucose variability measures were not included. Glucose variability is a common problem in patients with T1DM and is often associated with severe glucose excursions, and keeping in mind the fact that CHOC might lower the variability makes it an area that needs further investigation.

## 5. Conclusions

In most studies done in adults with T1DM using CHOC as a tool to calculate prandial insulin doses, glucose control was better than those using the usual care. This effect was observed in short-term periods (three months) and also in long-term periods, indicating the durability of the effectiveness. Therefore, CHOC is a useful tool, and preferably, all patients with T1DM should learn how to apply it starting at the diagnosis of the disease. Furthermore, the safety of the CHOC approach was established in most studies as there has not been an increased risk of hypoglycemia or weight gain. Moreover, the insulin doses did not show to be different in both groups, further supporting the safety margin of carb counting. Fortunately, technological advances have consolidated this effect, as CHOC mobile phone applications also show promising results in reducing HbA1c in a similar pattern.

However, in this review, no major difference in glucose control was appropriated in the pediatric age group, which might be related to the difficulties in controlling the diet in children.

As for the QoL and despite the multiple questionnaires used, better outcomes were observed in diabetes subjects using CHOC compared with those using standard care. More studies with higher numbers of patients are required to further demonstrate the efficacy of carb counting in reducing glucose excursions. Furthermore, local studies in the KSA are needed to address this issue in our population, keeping in mind the different types of food and dietary habits.

Moreover, in this review, only four studies were conducted using CHOC apps. In fact, using technology in nutrition, in general, is an evolving science and soon going to be a standard of care. Therefore, future work should focus more on using technologies such as image recognition and artificial intelligence. Because there is a strong tendency towards using continuous glucose monitoring systems and ambulatory glucose profiles as a way of measuring glucose control at the expense of HbA1c, more research is mandated to demonstrate the effect of CHOC across a time range [39,40]. 

## Figures and Tables

**Table 2:** Frequency of severe hypoglycemia.

Author/Year	Outcome	Definition of Hypoglycemia	Result
Adult			
Hommel E. et al., 2016 [25]	Episodes of severe hypoglycemia	Less than 3.9 mmol/L	Not significant, *p*-value not reported
Schmidt et al., 2012 [16]	Frequency of hypoglycemia	Self-reported (scored 0–6, perceived frequency is higher with higher scores)	Comparing control with CarbCount and CarbCountABC group (1.8 ± 1.4, 2.2 ± 1.1, 1.6 ± 1.2, *p* = 0.197) ^1^
Laurenzi et al., 2011 [26]	Frequency of hypoglycemia and episodes of severe hypoglycemia	≤2.8 mmol/L requiring assistance from a third party	Not significant, no severe hypoglycemia events reported
Scavone et al., 2010 [27]	Number of hypoglycemia events	Blood glucose < 3.9 mmol/L	Less hypoglycemic events in the CHOC group vs. control group (4% vs. 7%), *p* < 0.05
Trento et al., 2011 [28]	Severe hypoglycemic episodes	Hypoglycemia episodes requiring third-party help	CHOC vs. control (5 vs. 6 episodes), *p*-value not reported
Isaksson S. et al., 2021 [24]	The number of self-reported hypoglycemic events per month	Defined as glucose levels below 3.5 mmol/L	CHOC vs. control (0.05 vs. 0.07 events per month, *p* = 0.437)
Children and adolescents			
Enander et al., 2012 [31]	Frequency of hypoglycemia	Defined as plasma glucose < 3.5 mmol/L	Compared with baseline, hypoglycemia episodes in control, manual CHOC, and CHOC with a bolus calculator significantly reduced after intervention (*p* = 0.011) with no significant differences between groups
Donzeau A. et al., 2020 [32]	Episodes of severe hypoglycemia	Coma and/or convulsion	Intervention vs. control (3% vs. 2% patient/year, *p* < 0.05)

^1^ Data are mean ± standard deviation.

## Data Availability

Not applicable.
